# The perceptual consequences and neurophysiology of eye blinks

**DOI:** 10.3389/fnsys.2023.1242654

**Published:** 2023-08-16

**Authors:** Shawn M. Willett, Sarah K. Maenner, J. Patrick Mayo

**Affiliations:** ^1^Department of Ophthalmology, Center for the Neural Basis of Cognition, University of Pittsburgh, Pittsburgh, PA, United States; ^2^Department of Bioengineering, University of Pittsburgh, Pittsburgh, PA, United States

**Keywords:** vision, blinks, eyelid, eye movements, perception, corollary discharge, neuronal suppression, perceptual stability

## Abstract

A hand passing in front of a camera produces a large and obvious disruption of a video. Yet the closure of the eyelid during a blink, which lasts for hundreds of milliseconds and occurs thousands of times per day, typically goes unnoticed. What are the neural mechanisms that mediate our uninterrupted visual experience despite frequent occlusion of the eyes? Here, we review the existing literature on the neurophysiology, perceptual consequences, and behavioral dynamics of blinks. We begin by detailing the kinematics of the eyelid that define a blink. We next discuss the ways in which blinks alter visual function by occluding the pupil, decreasing visual sensitivity, and moving the eyes. Then, to anchor our understanding, we review the similarities between blinks and other actions that lead to reductions in visual sensitivity, such as saccadic eye movements. The similarity between these two actions has led to suggestions that they share a common neural substrate. We consider the extent of overlap in their neural circuits and go on to explain how recent findings regarding saccade suppression cast doubt on the strong version of the shared mechanism hypothesis. We also evaluate alternative explanations of how blink-related processes modulate neural activity to maintain visual stability: a reverberating corticothalamic loop to maintain information in the face of lid closure; and a suppression of visual transients related to lid closure. Next, we survey the many areas throughout the brain that contribute to the execution of, regulation of, or response to blinks. Regardless of the underlying mechanisms, blinks drastically attenuate our visual abilities, yet these perturbations fail to reach awareness. We conclude by outlining opportunities for future work to better understand how the brain maintains visual perception in the face of eye blinks. Future work will likely benefit from incorporating theories of perceptual stability, neurophysiology, and novel behavior paradigms to address issues central to our understanding of natural visual behavior and for the clinical rehabilitation of active vision.

## What is a blink and what is its function?

Blinks are a rapid and transient closure of the eyelid. They typically occur unconsciously, roughly 15 times per minute (Riggs et al., 1981; Volkmann et al., 1982; Evinger, 1995; Ousler et al., 2014; Van Opstal et al., 2016), and occur for many reasons, such as protecting the sensitive eyeball from damage and preventing the surface of the cornea from drying (Doane, 1980). Blinks are modulated by task demands and emotional state. Consequently, there are several types of blinks including: *reflexive blinks*, which occur unconsciously in response to an outside stimulus; *voluntary blinks*, which occur consciously; and *spontaneous blinks*, the most common type of blink, which occur unconsciously and are not typically evoked in response to stimuli (Stern et al., 1984; Evinger et al., 1991).

**FIGURE 1:**
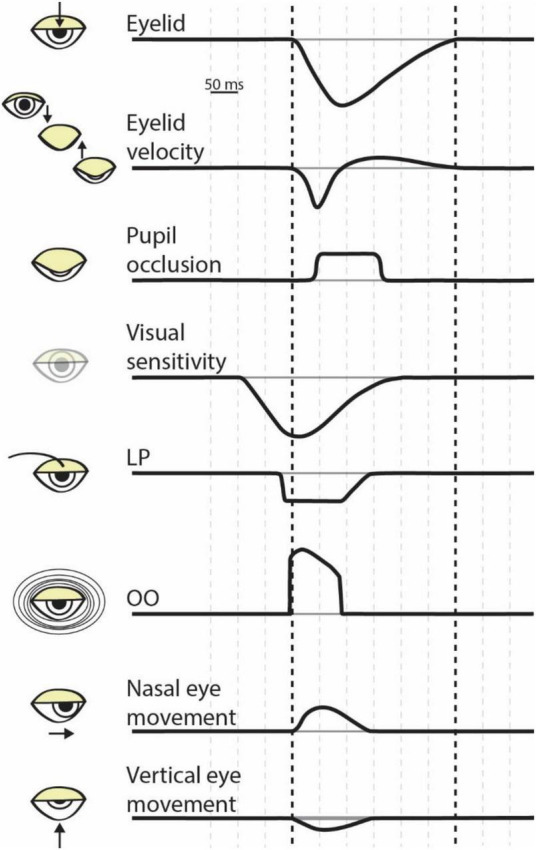
The dynamics of eye blink physiology. Each row illustrates the approximate timing of a component of a blink. The bolded vertical dashed lines correspond to blink onset (i.e., the initiation of eyelid closure) to blink offset (i.e., the return of the eyelid to the pre-blink fixation) as shown in the top row. Space between vertical dashed lines represents 50 ms. LP, levator palpebrae; OO, orbicularis oculi.

The different types of blinks have many similarities, from the muscles and movements involved to general kinematics. A blink physically begins with inhibition of the levator palpebrae (LP) muscle, which innervates the upper eyelid and maintains it in an open position (Stern et al., 1984; Dartt et al., 2011). Subsequently, the orbicularis oculi (OO) muscle contracts, rapidly pulling the upper eyelid down over the eyeball (Figure 1; Evinger et al., 1991). Simultaneously, the lower eyelid begins to move 3–5 millimeters in a nasal horizontal direction (Doane, 1980). All blinks have two phases: the “down phase” in which the upper eyelid rapidly descends, and the “up phase” in which the LP contracts and retracts the eyelid. The down phase is roughly twice as fast as the up phase, lasting roughly 75–100 milliseconds (Evinger et al., 1991). There are further nuances within these phases; the upper eyelid initially descends slowly via ligamental forces before rapidly accelerating to its peak velocity of 16–19 cm/s as it crosses the visual line, where it begins to slow before it meets the lower lid (Doane, 1980). The relationship between the amplitude of a blink and its peak velocity is linear, positive, and relatively constant, while the relationship between amplitude and duration is more variable because of the different speeds of the phases (Evinger et al., 1991; Costela et al., 2014). The up phase is generally slower because the LP must overcome passive orbital forces, resulting in up phases that last longer than the down phase but move the eyelid the same amplitude. These phases and kinematics are consistent regardless of the type of blink that is occurring.

The type of blink affects its velocity and timing. The down phase of reflex blinks is the fastest, while voluntary blinks are slower and more similar to spontaneous blinks which are the slowest (Evinger et al., 1991, though see Doane, 1980; Stern et al., 1984). It is thought that this is because spontaneous blinks are not evoked by a particular stimulus and therefore there is no need to rapidly close the eye lids. In contrast, reflex blinks are made when trying to protect the eye from an outside danger, so the eye quickly snaps shut. Once the danger has passed, the eyelid behaves as normal, such that the up phases of reflex, voluntary, and spontaneous blinks do not differ in velocity (Evinger et al., 1991). Thus, the closing of the eyelid can be used to characterize eye blink subtypes, while the up phase remains relatively constant across subtypes.

From inception to completion, blinks operate on the order of hundreds of milliseconds. The activation of the OO begins the rapid closure of the eyelid, and the lid begins to close around 10 ms afterward (Evinger et al., 1991). The down phase lasts between 50 and 130 ms (Stern et al., 1984). Full lid closure can last 10–50 milliseconds (Ousler et al., 2014, though see Doane, 1980), or longer when associated with a voluntary blink (Doane, 1980) or dry eyes (Ousler et al., 2014). In all, occlusion lasts ∼40–200 ms (Riggs et al., 1981; Volkmann et al., 1982); a significant period of time (Figure 1).

The duration of occlusion caused by a blink should, according to basic principles, lead to dramatic disturbances of the visual field. For example, an object passing through the visual field of a camera sensor for 100 ms would be easily noticed. Yet blinks are rarely noticed despite occurring thousands of times per day. Therefore, it is likely that there are brain mechanisms triggered by blinks which prevent them from reaching awareness. While the neural mechanisms of perceptual constancy for other visual-motor interruptions have long been studied (Von Helmholtz, 1925; Crapse and Sommer, 2008; Sommer and Wurtz, 2008), the mechanisms that mediate the somewhat simpler process of visual occlusion by eyelid closure remain mysterious. This review aims to summarize the current literature on perceptual constancy and the neurophysiology behind eye blinks. We will summarize the basic neurophysiological processes that underly blinks as well as the primary theories regarding their influence (or lack thereof) on visual perception. We hope that our work will help future researchers find and utilize the resources to investigate these processes.

## How do blinks affect perception?

Blinks interact with the brain’s perceptual machinery in three ways. First, blinks lead to a physical occlusion of the pupil by the eyelid. Second, they lead to a decrease in visual sensitivity independent of eyelid occlusion. Finally, they lead to stereotyped eye movements. Thus, blinks induce a wide range of sensory and motor processes that broadly impact neuronal activity and behavior.

The most obvious influence of blinks on perception is the eyelid’s occlusion of the pupil. Although the full process of a blink can last 250–450 milliseconds (Volkmann et al., 1982), the “blackout” caused by lid obstruction occurs for only 40–200 milliseconds (Doane, 1980; Volkmann et al., 1980, 1982). During this time, there is a roughly 100-fold reduction in light entering the eye (Riggs et al., 1981, though see Crawford and Marc, 1976). Therefore, around every 4 s a blink reduces full-field luminance and eliminates the ability to see for about 100 milliseconds. These intermittent disruptions in visual input are likely a fundamental part of how our brains process visual stimuli. For example, perception of an ambiguous stimulus such as the Necker Cube is stabilized by non-continuous presentation (Leopold et al., 2002; Ito et al., 2003; Nakatani and Leeuwen, 2005; Van Opstal et al., 2016). The disruption of visual input may also facilitate switching of percepts during binocular rivalry (Kalisvaart and Goossens, 2013). Lastly, pupil occlusion may help refresh the visual scene, in a manner similar to fixational eye movements (see Rucci and Casile, 2005; Martinez-Conde et al., 2006; McCamy et al., 2012). Somewhat paradoxically, eyelid occlusion therefore appears to be an important part of visual perception.

The effects of blinks are not a simple, direct consequence of eyelid occlusion. Blinks also appear to evoke a perceptual continuity system that allows for the brief moments of visual occlusion to go unnoticed. This is demonstrated by the fact that dimming of the entire visual field for a fraction of the blink duration (e.g., 30 milliseconds), like a flicker in the overhead lights (a much less intense stimulus than a blink), would be readily perceived (Moses, 1975; Riggs et al., 1981). One potential explanation for why these gaps in our perception are usually not noticed is because blinks decrease visual sensitivity. In a series of experiments, Volkmann et al. (1980, 1982) found that the ability to detect a change in luminance decreased up to five-fold, independent of lid occlusion of the pupil. This insight was cleverly demonstrated by using an apparatus to present a visual stimulus to the retina via the roof of the mouth. Volkmann et al. (1980) inserted a fiber optic bundle into the mouth of human subjects which illuminated the nasal portion of the retina in the right eye. Therefore, subjects perceived a diffuse cloud of light in their upper right visual field, regardless of whether their eyes were open or closed. The experimenters could then vary the luminance of the light field at different times relative to a blink and asked participants to indicate when a change in luminance occurred during a blink.

Volkmann et al. (1980) found that visual sensitivity to changes in luminance started decreasing *prior* to a voluntary blink, by about 100 milliseconds, and did not return to baseline levels until approximately 200 milliseconds after the blink onset (Figure 1). Importantly, the subjects performed this experiment in a dark room while wearing opaque goggles so that external light did not reach the retina. Therefore, a purely optical explanation of the results seems unlikely. Instead, the results suggest that that the change in visual sensitivity is caused by an active neural suppression of visual input during a blink (“blink suppression”). Follow up work showed this effect for reflex blinks (Manning et al., 1983) and that blink suppression is robust to light wavelength (Ridder and Tomlinson, 1995), but depends on spatial frequency (Ridder and Tomlinson, 1993, 1997). The visual suppression of a blink is extensive, so much so that for a stimulus to be subjectively equivalent to a voluntary blink the stimulus must have a four- to ten-fold weaker decrement in luminance and last for a significantly shorter duration than a blink (Riggs et al., 1981). In conclusion, blinks cause robust decreases in visual sensitivity across a range of stimuli and blink subtypes.

Blinks not only occlude the pupil and reduce visual sensitivity, but also induce stereotyped eye movements. The closure of the eyelid results in a downward rotation of the eye (Ongerboer de Visser and Bour, 2006). Regardless of the initial eye position, at blink onset the eye moves down and toward the nose before returning to the pre-blink fixation position (Figure 1). Although the start of the eye movement occurs at blink onset, the eyes return to fixation prior to blink offset. Initially, the movements were thought to be caused by the physical interaction of the eyelid with the eye (Doane, 1980; Volkmann et al., 1980). However, more recent work showed that the return-to-fixation movements accounted for consistent target displacements during blink, implying independent neuronal control (Maus et al., 2017). Any movement of the eye likely interacts with perception and blink-related eye movements are no exception. As mentioned above, these movements may help prevent visual fading; and they are known to elicit illusory motion (Otero-Millan et al., 2012). Importantly, blink associated movements may alter the efficacy of fixation. The eye movements associated with blinks have been shown to increase fixational errors and destabilize fixation (Costela et al., 2014) but in other contexts, may compensate for large errors in fixation (Khazali et al., 2017). Consequently, the movements of the eyes accompanying blinks influence fixation performance which, in turn, impact foveal vision.

Pupil occlusion, attenuation of visual sensitivity, and blink-related eye movements all likely play a significant role in natural visual behavior, to the degree that blink rate is highly regulated. The presence of a visual stimulus alone can significantly reduce the likelihood of blinking (Williamson et al., 2005). Subjects engaged in visual tasks drastically reduce their blink rates and preferentially blink in inter-trial intervals (Goldstein et al., 1985; Van Opstal et al., 2016). A high concentration of blinks in low information periods of experimental tasks is also observed during natural behaviors, where blinks preferentially occur at the end of reading a sentence or when turning a page (Hall, 1945; Orchard and Stern, 1991). The effects of blinks even extend beyond visual tasks; blink rate is also attenuated during auditory stimulus presentation and prior to an expected auditory target (Kobald et al., 2019; Abeles et al., 2020). Accordingly, it seems likely the nervous system regulates blink timing to ameliorate the deleterious effects of blinks on perception. Indeed, blinks synchronize with other behaviors known to impair perception such as saccades, combined eye-head movements (Zee et al., 1983; Evinger et al., 1994; Goossens and Van Opstal, 2000a), and other types of eye movements (Khazali et al., 2016). Interestingly, blinks and eye movements are so intertwined that blinks can initiate vergence eye movements to restore binocular vision in patients with limited binocular control (Jampolsky, 1970; Peli and McCormack, 1986; Hatt et al., 2009).

## Saccadic eye movements as a proxy for understanding blink-related perceptual stability

Blinks interrupt vision and engage extraretinal mechanisms that support visual stability. So, it may be useful to consider other visual-motor phenomena that require similar perceptual “filling in.” The rapid translation and visual smearing across the retina caused by saccadic eye movements may serve as a proxy for understanding the influence of blinks on visual perception. Saccades are rapid, redirections of the high-acuity fovea across the visual field that occur about three times per second. In addition to creating a blur across the retina, sensitivity to visual stimuli drops precipitously roughly 100 milliseconds prior to saccade onset and extends to 100 milliseconds after saccade onset (Diamond et al., 2000; Ross et al., 2001; Ibbotson and Krekelberg, 2011). Therefore, like blinks, saccades disrupt stable visual processing. Saccades and blink suppression exhibit many other commonalities: they share a sensitivity to spatial frequency (Ridder and Tomlinson, 1993, 1997; Diamond et al., 2000); have similar profiles within the same subject (Ridder and Tomlinson, 1997); and are thought to involve active neural suppression of visual input during their execution (Volkmann et al., 1980, 1982; Sommer and Wurtz, 2008). Indeed, it has been hypothesized that blink and saccade suppression share similar mechanisms (Volkmann et al., 1982; Ridder and Tomlinson, 1997). It is important to note, however, that the eye movements that result from blink may be unlikely to underlie the active component of blink suppression because they may result from the downward motion of the eyelid (Doane, 1980) and are known to not display typical saccadic kinematics (Volkmann et al., 1980; Costela et al., 2014; Khazali et al., 2016). Interestingly, saccades, like blinks, go unnoticed and typically fail to disrupt visual continuity. How, then, do blinks and saccades disrupt vision so significantly but fail to elicit movement-induced changes in perception?

Compared to blinks, there has been much more work investigating the neural mechanisms underlying visual stability during saccadic eye movements. Similarly, to blinks (see below), there is evidence that suppression of ongoing cortical activity may prevent awareness of saccadic eye movements. Specifically, some neurons in area MT/MST fail to respond to the saccade-evoked motion of the image across the retina and are suppressed by saccades (Thiele et al., 2002). This suppression may be accompanied by a transient shift in neuronal receptive fields. For instance, neurons in many cortical regions are known to shift their directional tuning or spatial sensitivity (i.e., receptive fields) in preparation for a saccade: areas MT/MST (Thiele et al., 2002; Womelsdorf et al., 2006); the frontal eye fields (Umeno and Goldberg, 1997; Sommer and Wurtz, 2006, for review see Rao et al., 2016); LIP: (Duhamel et al., 1992); V4: (Neupane et al., 2016); V2: (Nakamura and Colby, 2002; Denagamage et al., 2023). These shifts in neuronal selectivity are thought to facilitate visual stability and originate in part through corollary discharge signals. Indeed, inhibition of corollary discharge led to errors in the localization of targets (Cavanaugh et al., 2016). Ultimately, saccades and blinks generate large disruptions to vision, involve an active suppression of visual input during movement, and may rely on similar neuronal processes to maintain visual stability.

It is important to note that although there are many similarities between saccades and blinks, there are also stark differences between the two movements. For example, both blinks and saccades suppress cortical activity, yet the underlying suppressive mechanisms are likely different. Blink-related cortical suppression is assumed to rely entirely on corollary discharge signaling eyelid closure (Volkmann et al., 1980, 1982). While this may also be the case for saccades (Sommer and Wurtz, 2008), more recent work has proposed that saccade-induced suppression could result from a purely visual mechanism. That is, cortical suppression may be inherited from the retina; saccade-induced image translations result in attenuation of photoreceptor activity that is then carried throughout the visual system (Idrees et al., 2020; Baumann et al., 2021). Alternatively, saccade-induced cortical suppression may be due to a sublinear integration of the corollary discharge signal and the incoming visual information (Miura and Scanziani, 2022). It should also be noted that the corollary discharge signal for saccades and blinks likely originates in different nuclei, the superior colliculus for saccades (Sommer and Wurtz, 2002, 2006) and the facial or oculomotor nucleus (Manning et al., 1983) for blinks. Furthermore, it seems that the saccade induced modulation of cortical activity also has a directional component because saccades transiently altar the direction tuning of neurons (Thiele et al., 2002) or shift spatial receptive fields in the same direction of the eye movement (Duhamel et al., 1992; Sommer and Wurtz, 2006). This remapping also occurs in the attentional system, which allows for attended locations to remain stable while preventing blur and motion signals generated by the saccade to reach awareness (see Mirpour and Bisley, 2015). The blink command is one dimensional and therefore, spatial or attentional remapping cannot explain visual stability across blinks. Lastly, saccades are accompanied by a compression of space. Around the time of saccades subjects mis-localize objects, underestimating their distance to the saccade endpoint (for review see Ross et al., 2001; Haladjian et al., 2015); and similar spatial compression changes have also been reported in the receptive fields of cortical neurons (Tolias et al., 2001; Zirnsak et al., 2014; Neupane et al., 2016). However, blinks do not elicit spatial compression (Haladjian et al., 2015). Overall, there are many similarities and differences between the neural cascade induced by a blink or a saccade, and future work will need to disentangle these two processes.

## What are the critical brain regions for controlling blinks?

There are two major hypotheses regarding how the visual system maintains continuity in the face of blinks: recurrent activity and suppression of visual transients. The first hypothesis proposes that recurrent neuronal activity generated via corticothalamic loops could underlie a “filling in” process (Billock, 1997). The idea centers around an anatomical loop between primary visual cortex (V1) and the lateral geniculate nucleus that becomes active at the onset of a blink. Activation of this processing loop maintains information concerning visual stimuli prior to pupil occlusion and effectively “fills in” the visual scene. Corticothalamic loops are powerful architectures known for their involvement in several cognitive functions including maintenance of persistent cortical activity (Guo et al., 2017). Although V1 contains much information about the visual scene, it may be more efficient to have a higher order cortical area, one whose activity is more correlated to conscious perception, supply input to the thalamus. In response to a blink, neurons in the putative “fill in” cortical area would enhance their firing rate. This hypothesis has received some support as demonstrated by parieto-occipital cortex activation around time of blinks (Bristow et al., 2005a; Guipponi et al., 2014). Thus, it is plausible that a corticothalamic loop could maintain memory of the visual scene throughout a blink.

The second hypothesis predicts a suppression of visual transients that is time-locked to blink onset. This theory relies on the notion that visual stimuli that reach awareness do so because they evoke a significant neuronal transient response. If those transients are prevented from occurring through suppression, then those stimuli will not reach perception. The neuronal suppression could rely on a corollary discharge signal from the motor nucleus responsible for the blink and attenuate ongoing activity in downstream areas. This theory, also, has garnered empirical support. For instance, some neurons in monkey visual areas V1, V2, V3V/VP, and V4V are suppressed around the time of blink (Gawne and Martin, 2000, 2002). There are additional reports of areas in occipital, parietal, and frontal cortices that show blink related suppression (Bristow et al., 2005b). Because both hypotheses have garnered preliminary empirical support, it is possible that the nervous system implements both methods to reduce the disruptions of blinks on visual perception.

**FIGURE 2:**
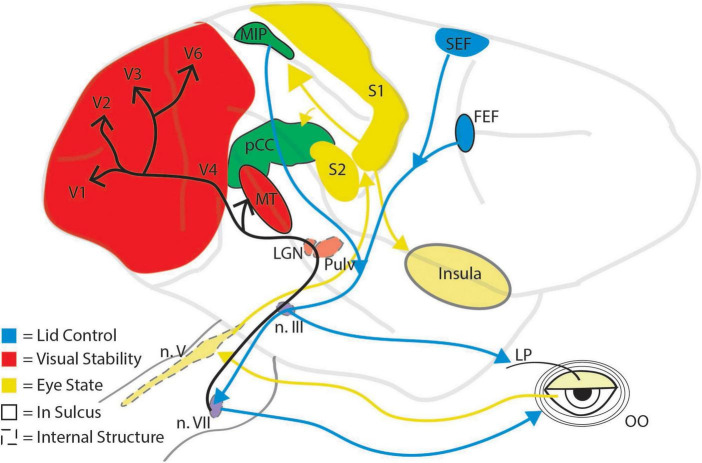
Schematic illustration of major brain regions implicated in the control of and response to eye blinks. Brain regions are grouped by color according to their primary role in eye blinks: red, regions involved in visual stability; yellow, regions involved in representing the state of the eye, and blue, regions involved in initiating blinks. Mixed colors (green and purple) indicate brain areas that have dual functions. Dashed outlines indicate subcortical structures, solid outlines indicated structures within a sulcus, and arrows represent processing pathways. Pulv, pulvinar; LP, levator palpebrae; OO, orbicularis oculi; n. III, oculomotor nucleus; n. V, trigeminal nucleus; n. VII, facial nucleus.

The maintenance of visual activity for perceptual filling in and the inhibition of visual transients can manifest in a variety of neuronal mechanisms and brain regions. Given that blinks can be reflexive, spontaneous, or voluntary and that they alter eye movements and perception, it is unsurprising that multiple cortical and subcortical brain areas contribute to the control of and response to blinks (Figure 2). The neural circuitry responsible for blinks largely converges on two neuronal populations that regulate the closure of the eyelid: the motor neurons of the orbicularis oculi (OO) located in the facial nucleus (May and Warren, 2021), and the motor neurons of the levator palpebrae (LP) located in the oculomotor nucleus (Rucker, 2010; Warren and May, 2021). Not only are these neuronal populations involved in the closure of the eyelid, but they are also the likely source of the corollary discharge signal used to decrease visual sensitivity during a blink. Because blink-related visual suppression occurs even in response to reflexive blinks (Manning et al., 1983), it is expected that the corollary discharge source is situated lower in the brainstem and closer to the motor output, as opposed to the superior colliculus, which is thought to produce saccadic corollary discharge (Sommer and Wurtz, 2002, 2008). Therefore, the OO and LP motor neurons are prime candidates. The response dynamics of the OO motor neurons resemble those of established corollary discharge signals. The superior colliculus corollary discharge signal is a strong burst associated with the rapid translation of the eye, and, similarly, the OO motor neurons display a strong burst of neuronal activity associated with the rapid closure of the eyelid (Trigo et al., 1999). In contrast, the LP motor neurons are transiently inactivated around the time of a blink and show stronger activity during the opening movement of the eyelid, around the time which visual sensitivity is recovering (Figure 1). Future work will need to elucidate which brainstem nuclei contribute to blink associated corollary discharge.

Even though the precise origin of the corollary discharge signal is unknown, there is at least some research investigating the neurobiological underpinnings of across-blink visual stability. Early work in the rhesus macaques found that some primary visual cortex (V1) neurons were suppressed more when the monkey blinked compared to when the external scene was darkened (Gawne and Martin, 2000). Follow up work examined neurons in areas V2, V3, and V4 and found a similar result, although blink-modulated neurons were a minority in the total population of neurons studied (Gawne and Martin, 2002). However, this small population of neurons throughout the visual cortex responded differently if a luminance change was caused by a blink versus by external darkening (Gawne and Martin, 2002) and could play a critical role in shaping perception around the time of blinks.

A major limitation of early neurophysiological studies was that they were unable to present a stimulus to the retina during blinks. Therefore, the results could simply be due to subtle differences in luminance changes in blinks compared to those during external darkening. Though accompanied with their own methodological constraints (see below), more recent human functional imaging studies attempted to address this confound by presenting light to the retina independent of the pupil (Bristow et al., 2005b), through a fiber optic cable in the mouth (similar to Volkmann et al., 1980). Although many of the regions in the early stages of visual processing (lateral geniculate nucleus through V3) showed less activity during voluntary blinking, only area V3 showed a significant reduction (Bristow et al., 2005b). Intriguingly, the authors only found suppression if retinal stimulation was present; in conditions without retinal stimulation blinks *increased* activity throughout the visual pathway. Other work from the same researchers found a greater degree of suppression during blinks than during external darkening throughout the visual cortex including area MT. However, they found blink-induced *activation* in a medial parietal-occipital region, homologous to macaque area V6 (Bristow et al., 2005a). The authors suggest that suppression in early visual areas explains the loss of visual sensitivity while activation in higher visual areas maintains visual continuity (Bristow et al., 2005a).

Bristow et al. (2005a,b) asked subjects to continuously blink, resulting in blink rates of over 130 blinks per minute, far larger than the blink rates during natural visual behavior. The extreme blink rates may have led to atypical responses throughout the brain. More recent work that looked at activity after a blink found neural activation, not suppression, in early visual cortical areas (Hupé et al., 2012; Guipponi et al., 2014). Although task differences could be important, the discrepancy across studies may originate from the fact that only a small population of neurons carry information concerning self-generated (blink) versus externally-generated (darkening) luminance changes (Gawne and Martin, 2002). This population may be too small to reliably detect using functional neuroimaging. Nevertheless, it appears that low- to intermediate-level visual cortex and parietal-occipital areas may be key structures to investigate to understand across-blink visual stability.

Through what pathways might the corollary discharge signal reach cortex from the brainstem? In the saccadic system, corollary discharge signals emerge from the superior colliculus, synapse within the medial dorsal nucleus of the thalamus, and then ascend to cortex (Sommer and Wurtz, 2002, 2008; Berman et al., 2009; Wurtz et al., 2011). If we assume that blink suppression anatomy is similar to most other ascending brainstem signals (e.g., saccade suppression), the corollary discharge signaling blink would be expected to synapse within the thalamus before ascending into cortex. Two candidate thalamic nuclei are the lateral geniculate nucleus and the pulvinar nucleus. There is some evidence that the lateral geniculate nucleus is modulated by blinks (Bristow et al., 2005b), and it is theorized to play a role in maintaining visual experience during blinks (Billock, 1997). Likewise, the pulvinar is known to connect to and modulate many visual cortical areas (Berman and Wurtz, 2011; Saalmann et al., 2012; Eradath et al., 2021; Miura and Scanziani, 2022), but it is not known if pulvinar neurons alter their activity during blinks.

Another neurophysiological consideration is: what areas control *when* we blink? The current state of the eye exhibits a strong influence on the blink circuitry. Reflexive blinks, for example, occur when trigeminal afferents from the cornea or eyelid are activated by a physical perturbation to the eye. These afferents have their cell bodies in different parts of the trigeminal nucleus and mono-synaptically innervate OO and LP motor neurons (May and Warren, 2021; Warren and May, 2021). The same afferents also play a significant role in spontaneous blinks. Unlike reflexive blinks, the neural mechanisms of spontaneous blinks extend well beyond brainstem nuclei. For example, spontaneous blinking in macaque monkeys is governed by a core somatosensory network involving primary somatosensory cortex, secondary somatosensory cortex, the parietal median intraparietal areas (MIP), cingulate, and insula (Guipponi et al., 2014). The authors posit that the prolonged fixation requirements of their task could lead to corneal and eye strain, which would activate this core somatosensory network through the trigeminal afferents and eventually engage higher cortical areas that represent awareness of the need to blink (Lerner et al., 2008; Guipponi et al., 2014). This core somatosensory network would then drive activity in the cingulate eye fields or the MIP, areas known to contain oculomotor activity (Olson et al., 1996), connect to OO motor neurons (Gong et al., 2005), and evoke blinks when stimulated (Thier and Andersen, 1998). Perhaps unsurprisingly, other cortical eye fields such as the supplementary eye fields (SEF) and the frontal eye fields (FEF) likely help regulate when a blink is initiated. Both the SEF and FEF are thought to control voluntary blinking and are activated in numerous functional neuroimaging studies investigating voluntary and spontaneous blinks (Kato and Miyauchi, 2003; Bristow et al., 2005b; Hupé et al., 2012; Guipponi et al., 2014). Lastly, it is important to note that changes in blink rate are also affected by dopaminergic tone. Parkinson’s disease patients have lower blink rates than controls, while patients with higher dopamine modulation such as in Schizophrenia display higher than typical blink rates (Karson, 1983). How dopaminergic centers like the ventral tegmental area (VTA) and the basal ganglia interact with the motor neurons controlling eyelid movement remains unclear and may have important diagnostic implications for dopamine-related disease states. Although blinks are a simple movement, they significantly alter visual processing which requires much of the brain to limit their impact on perception and to regulate their occurrence during behavior.

A final topic of interest is the neural circuits that allow timing and kinematics of reflex blinks to adapt. These processes are known as eye blink conditioning and blink adaptation, respectively. Although these processes seem to involve many other brain structures (Parras et al., 2022), the cerebellum is recognized as an important region for eye blink motor learning, and eye blink conditioning specifically has been used to study the bases of motor learning and memory. Eye blink conditioning involves the pairing of a predictive stimulus (usually an audio tone) with a stimulus that triggers a blink (e.g., an air puff). Over many pairings of these stimuli, the subject will perform a conditioned blink in response to the predictive stimulus to avoid the air puff. That is, the timing of their blink will shift earlier in time so that the eye lid is closed during the air puff. Modulations of the cerebellar purkinje cell activity through mossy fiber (predictive stimulus) and climbing fiber (blink response) inputs is thought to underlie this behavior, with additional processing in the deep cerebellar nuclei, red nucleus, and the cranial motor nuclei that control lid closure (Thompson and Krupa, 1994; Attwell et al., 2002; Christian and Thompson, 2003; Gerwig et al., 2007). Although the cerebellum seems to play a role in unconditioned reflexive blink generation (Pellegrini and Evinger, 1997), conditioned blinks have wildly different kinematics–a lid closing velocity of roughly a tenth of a reflexive blink (Trigo et al., 1999). This difference in behavioral metrics implies that the cerebellar-dependent conditioned blinks are generated by unique neural circuitry (see Parras et al., 2022). Unlike in blink conditioning, which results in a blink in response to a typically non-blink-evoking stimulus, blink adaptation results in a modulation of the motor response to a stimulus that typically evokes a blink. For example, after many blinks paired with a weight on the eyelid the response of the orbicularis oculi will decrease (Evinger and Manning, 1988). Blink adaptation is also thought rely on the cerebellum, specifically populations of neurons within the interpositus nucleus (Chen and Craig, 2006) and cerebellar cortex (Pellegrini and Evinger, 1997). While more work is needed to investigate the role of cerebellum in unconditioned reflexive blinks, the cerebellum seems tightly coupled to the neural populations responsible for generating conditioned eye blinks.

## Open questions

The simple motor act that defines a blink belies the richness of the sensory-motor processes that accompany them. To better understand eye blinks and their impact on brain activity, we have divided blink-related neural mechanisms into three categories. First, blinks are elicited by a constellation of voluntary, cognitive, and reflexive processes that monitor and modulate the current state of the eye. Second, blinks engage motor circuitry proximal to the eyes that not only activate eyelid muscles but also typically trigger concomitant changes in the eye muscles to generate eye movements. Finally, the pupil occlusion associated with blinks typically goes unnoticed, suggesting a precise interplay of eyelid motor commands and visual mechanisms for perceptual continuity. Thus, the “simple” act of an eye blink elicits a wide range of sensory, cognitive, and motor components across a broad range of neural circuits.

Despite the multitude of ways in which blinks affect vision and the ubiquity of blinking in our daily lives, there has been little work on the neurophysiological mechanism that support these processes. This knowledge gap contrasts with decades of in-depth research on the related mechanisms of visual stability around the time of rapid eye movements. While many studies on eye blinks have capitalized on functional MRI to measure brain-wide changes in blink-related hemodynamic signals (Kato and Miyauchi, 2003; Bristow et al., 2005a,b; Hupé et al., 2012; Guipponi et al., 2014), extracellular neuronal recordings during and around the time of blinks in primates are rare (Gawne and Martin, 2000, 2002; Goossens and Van Opstal, 2000b). The precision afforded by such recordings will likely be necessary to disentangle the rapid modulation of visual-motor signals during blinks, alongside precise behavioral measurements and simultaneous recordings across brain regions. A candidate mechanism for visual stability is corollary discharge (or, efference copy) and corollary discharge, by its very nature, involves rapid communication between sensory and motor brain areas. Although blink-related suppression of visual transients may help mitigate sensory changes caused by blinks, a predictive, compensatory mechanism like corollary discharge seems likely to play a role.

Given that research on the neural mechanisms of blinks is still in its infancy, the most pressing open questions regarding the neural mechanism of eye blinks are perhaps the most basic. They can be broken down into conceptual questions and technical challenges. Conceptually, what are the neuronal mechanisms that mediate uninterrupted vision despite fully occluded pupils, and how do these mechanisms affect ongoing computations in visual cortex? Technically, how do we test visual function in the eye when the eyelid is closed (Volkmann et al., 1980, 1982; Riggs et al., 1981; Manning et al., 1983)? Ideally, we would like to present high resolution images to the retina while the eyelid is open versus closed, but this approach requires significant technological development. Are blink related changes in perception unique to vision? This seems unlikely given sounds can evoke reflexive blinks (Hori et al., 1986) and alter blink rate (Kobald et al., 2019; Abeles et al., 2020); yet work on blink related changes to audition is sparse. Nevertheless, these avenues for future work are tractable and–in light of the tens of thousands of blinks we perform daily–represent a promising way to understand the fundamental principles of brain function.

## Author contributions

SW, SM, and JM designed the research and wrote and revised the manuscript. SW and SM performed the research. All authors contributed to the article and approved the submitted version.
